# Intraoperative Fluorescein Angiography Can Efficiently Identify Biomarkers and Guide Surgical Decision-Making

**DOI:** 10.1097/IAE.0000000000003790

**Published:** 2023-11-17

**Authors:** Michael S Cardamone, Gustavo Hüning, Chadd Scarlett, Lu Yin, Alexander J. Luna, Alan J. Franklin

**Affiliations:** * Alcon Laboratories, Fort Worth, Texas; † Hüning Clínica do Olhar., Santa Maria, RS, Brazil;; ‡ University of Toledo College of Medicine and Life Sciences, Toledo, Ohio; § Diagnostic and Medical Clinic, Mobile Infirmary Medical Center, Mobile, Alabama.; Reprint requests: Alan Franklin, MD, PhD, Diagnostic and Medical Clinic, 1720 Springhill Avenue, Suite 300, Mobile, AL 36604; e-mail: Alfranklin84@gmail.com

**Keywords:** vitrectomy, surgical visualization, fluorescein angiography, surgical technique

## Abstract

**Purpose:**

We sought to develop an efficient method for fluorescein angiography (FA) during digitally assisted vitreoretinal surgery (DAVS).

**Methods:**

A 485-nm bandpass filter was placed into the filter holder of the accessory light sources of the Constellation Vision System with steel modified washers to produce an exciter source. A barrier filter was placed into the blank slot of a switchable laser filter with a 535-nm bandpass filter and another washer or created digitally with a specific color channel using NGENUITY software version 1.4. Fluorescein, 250 to 500 mg, was then injected intravenously during retinal surgery.

**Results:**

These fluorescence patterns accurately detect many fluorescein angiography biomarkers, such as determination of vascular filling times, ischemia, neovascularization, shunt vessels, microaneurysms, and leakage into the vitreous. This enhanced surgical visualization permitted intervention in real time such as laser or diathermy to residual microvascular abnormalities after delamination of retinal neovascularization as well as heavier panretinal laser placement in areas of retinal capillary dropout to relatively preserve areas of more intact retinal microcirculation.

**Conclusion:**

The authors of this study are the first to report an efficient method that permits high-resolution detection of many classic FA biomarkers such as during DAVS to enhance surgical visualization and intervention in real time.

## Abstract 2

Supplemental Digital Content is Available in the Text.

This article reports an efficient intraoperative fluorescein angiography method that permits high-resolution detection of many classic FA biomarkers, such as vascular filling time, neovascularization, capillary dropout, shunt vessels, microaneurysms, and vascular leakage during digitally assisted vitreoretinal surgery to enhance surgical visualization and intervention in real time.

Fluorescein angiography has been a cornerstone in the diagnostic armamentarium for retinal diseases since the 1960s.^1^ Typically, fluorescein angiography is used in the clinic to diagnose a host of vascular, inflammatory, and genetic pathologies. Intraoperative fluorescein angiography (IOFA) was first reported by Charles^2^ in the 1980s. Subsequently, Avery and others have had success with the technique.^3^ However, the captured images were limited by both resolution and two dimensionality secondary to the optical visualization technology.^4–7^ Moreover, under the conventional optical surgical microscope systems, surgery was difficult to perform while implementing intraoperative FA.

The use of a high-definition three-dimensional screen was first developed in 2008, where it was used for anterior segment surgery.^8^ As cameras and digital imaging processing became more advanced, digitally assisted vitreoretinal surgery (DAVS) was refined several years later for both anterior segment and posterior segment procedures. Initially, the camera resolution, digital image quality, and latency of the feed limited this platform for surgical visualization. However, as digital technology has evolved over the past 5 to 7 years, the current iteration of DAVS has several advantages over traditional microscopic systems that include enhanced depth of field, depth of resolution (stereopsis), better performance in low-light environment, real-time visualization of the retinal machine function and fluidics, and the ability to use digital filters to enhance the surgical image in real time.^9–13^

Imai et al^14^ have developed IOFA during DAVS for pars plana vitrectomy (PPV) and reported the treatment of proliferative diabetic retinopathy (PDR). We have modified the method of Imai, by placing the exciter filter directly into the Constellation Vision System (CVS) to avoid switching to an alternative light source and ultimately designing a digital barrier filter for DAVS that omits the need for placement of an analog barrier filter in the surgical microscope. The update in this technique permits efficient transition to and from angiography visualization during DAVS and permits effective visualization of many fluorescein angiography biomarkers that include vascular filling times, microvascular blockage, retinal and choroidal neovascularization, and inflammatory-based leakage to enhance intraoperative surgical decisions.

## Materials and Methods

### Subjects and Methods

This was a single-site case series study. The study adhered to the tenets of the World Medical Association Declaration of Helsinki. Intraoperative fluorescein angiography with DAVS was performed in patients.

### Surgical Procedures

Surgeries were performed by two vitreoretinal surgeons in a single operative room, G.H. and A.F. The surgeon wore passive 3D polarized glasses and was positioned 1.2 m away from the screen. The NGENUITY 3D visualization system (Alcon Laboratories, Inc, Fort Worth, TX) was used as a platform for image processing. During standard PPV using the CVS (Alcon Laboratories, Inc) with either a wide-angle noncontact viewing system (Resight; Carl Zeiss Meditec AG, Jena, Germany) or a contact system (Miniquad, Volk, Mentor, OH) panretinal photocoagulation (PRP), removal of fibrovascular proliferative membrane (FVERM), removal of subretinal neovascular membrane (CNVM), and laser photocoagulation or diathermy to leaking vascular abnormalities were performed under IOFA. Subconjunctival corticosteroid and postoperative antibiotic ointments were administered at the end of the surgical procedure.

### Three-Dimensional Fluorescein Angiography Procedures

**Fig. 1.**
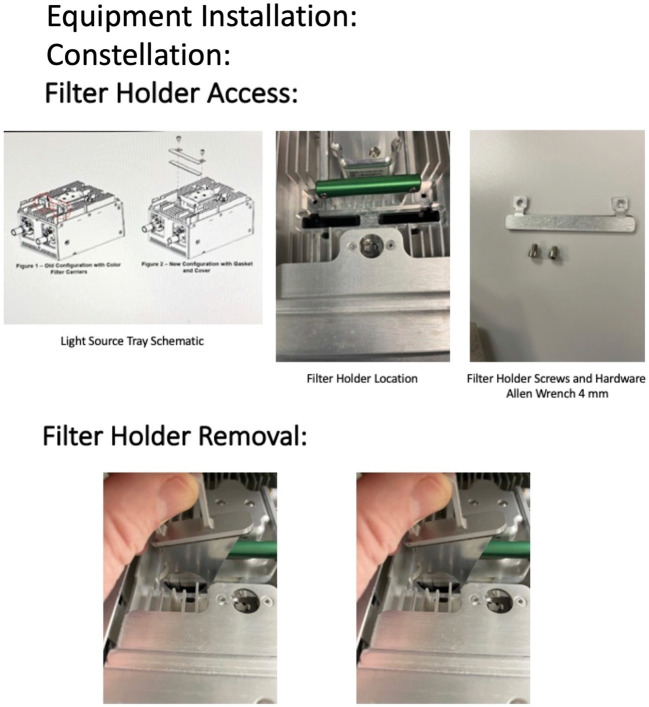
Preparation of the Constellation Vision System for IOFA. A 485-nm bandpass filter exciter was placed into the filter holder of the accessory light sources of the CVS with steel modified washers. The placement and removal of the filter holders are depicted.

**Fig. 2.**
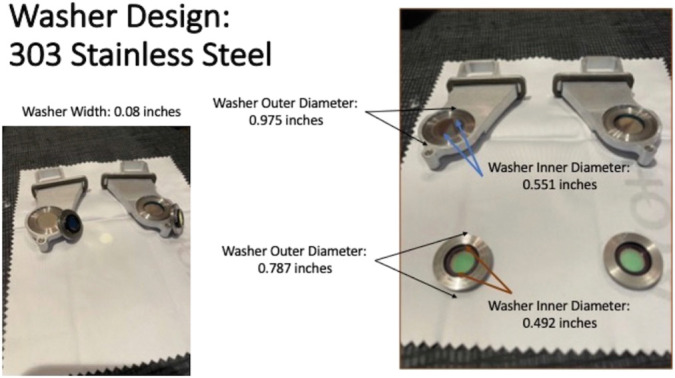
Assembly of the exciter and barrier filters. A 485-nm bandpass filter, 14 mm in diameter, was placed into the a steel washer 14 × 25 × 3 mm, and this washer was then placed inside the 25 mm filter holder to create an exciter filter. To fashion a barrier filter, a 535-nm bandpass filter was placed in another steel washer with an outer diameter of 20 mm, inner diameter of 12.5 mm, and width of 2.0 mm for placement into the blank slot of a switchable laser filter of the surgical microscope to pro- duce a barrier filter.

**Fig. 3.**
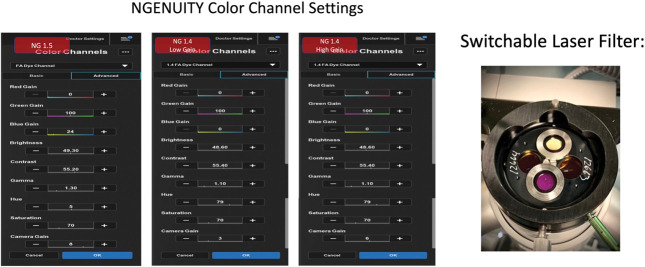
Creation of digital barrier filter and placement of the optical barrier filter. Initially, a 535-nm bandpass filter was placed in another steel washer with an outer diameter of 20 mm, inner diameter of 12.5 mm, and width of 2.0 mm to was placed into the blank slot of a switchable laser filter to produce a barrier filter. Ultimately, a specific color channel was developed for DAVS using NGENUITY for both software versions 1.4 and 1.5 to engineer a digital barrier filter. The specifications of these digital filters or color channels are shown where NG 1.4 is NGENUITY version 1.4 software and NG 1.5 is NGENUITY version 1.5 software.

A 485-nm bandpass filter, 14 mm in diameter, Semrock, IDEX Health and Science, LLC, Rochester, NY, was placed into the filter holder of the accessory light sources of the CVS with steel modified washers, Figure 1. The steel washers were engineered with an outer diameter of 24.77 mm, inner diameter of 14.00 mm, and width of 2.0 mm (Figure 2) to produce an exciter source. Initially, a 535-nm bandpass filter, 12.5 mm in diameter, Edmunds Optics, Barrington, NJ, was placed in another steel washer with an outer diameter of 20 mm, inner diameter of 12.5 mm, and width of 2.0 mm and was ultimately placed into the blank slot of an switchable laser filter to produce a barrier filter Figure 3. As an alternative to the physical barrier filter, a specific color channel was developed for DAVS using NGENUITY to engineer a digital barrier filter, Figure 3. To perform IOFA, a light pipe endoilluminator and/or a chandelier was placed into the accessory light source of the CVS, where the exciter filters had been installed and the light source intensities were increased to 100 for the light pipe and 105 for the chandelier. Sodium fluorescein, 2.5 to 5 mL, 100 mg/mL, Alcon Surgical, Ft. Worth, TX, was then injected intravenously to produce a signal.

## Results

### Vascular Filling Times

Vascular filling times were observed in real time. The choroidal phase is qualitatively more pronounced with relative increased fluorescence to that noted in the clinic. Increased filling times were observed in the settings of increased intraocular pressure, systemic hypotension, and poor microvascular circulation, Supplemental Video (http://links.lww.com/IAE/B966).

### Vascular Blockage

Discrete vascular blockages are readily identified in branch retinal vein occlusions. In the scenario of central retinal vein occlusions, increased vascular filling time, venous tortuosity, and arteriovenous shunt vessels can be identified, Supplemental Video (http://links.lww.com/IAE/B966).

### Microvascular Abnormalities

Multiple intravascular abnormalities can also be identified. Residual microvascular abnormalities with leakage can be found after membrane delamination for proliferative diabetic retinopathy or other ischemic retinopathies only with IOFA. Laser or diathermy can be applied to these residual microvascular abnormalities to decrease the risk of postoperative vitreous hemorrhage, Supplemental Video (http://links.lww.com/IAE/B966). Regions of retinal capillary dropout can be identified by IOFA so that the panretinal laser can be altered to treat areas of ischemia with more confluent laser to relatively spare areas of better perfused retina, Supplemental Video (http://links.lww.com/IAE/B966).

### Inflammatory-Based Leakage

Both inflammatory cystoid macular edema and perivascular leakage can be visualized in eyes affected by uveitis or infection, Supplemental Video (http://links.lww.com/IAE/B966). In addition, relative activity of inflammatory lesions can be observed by the presence or absence of fluorescein leakage.

### Neovascularization

Retinal neovascularization often adjacent to areas of retinal capillary dropout were easily observable during IOFA. Often, the delineation of neovascularization was helpful to identify tissue to delaminate during vitrectomy, Supplemental Video (http://links.lww.com/IAE/B966). Their high degree of depth of resolution permitted precise delamination of internal limiting membrane in some cases, Supplemental Video (http://links.lww.com/IAE/B966). Finally, CNVM could be identified and removed surgically, Supplemental Video (http://links.lww.com/IAE/B966).

## Conclusions

We were able to modify the technique reported by Imai by placing exciter filters directly into the CVS and ultimately developing a digital barrier filter to permit high resolution and efficient transition to and from IOFA visualization in real time during DAVS. The transition to IOFA and back to standard DAVS routinely takes less than 1 minute. This negligible delay intraoperatively allows IOFA to be routinely used without significantly increasing operative time. Intraoperative fluorescein angiography reliably reproduced many routine clinical biomarkers such as vascular filling times, vascular occlusions, shunt vessels, retinal capillary dropout, and both retinal and choroidal neovascularization.

Delay in vascular filling times was observed in the scenarios of increased intraocular pressure, systemic hypotension, or retinal ischemia. Each of these scenarios guided us to alter our surgical approach. In the case of poor vascular flow from increased intraocular pressure, we lowered the intraocular pressure from the CVS to permit better perfusion to the retina. Hayreh reported irreversible vision loss after 90 minutes or more of restriction of flow to the retina in healthy animals^15^ so that it is salient to note the effects of increased intraocular pressure on retinal circulation during complicated diabetic vitrectomy surgery. It is also important to visualize the effects of retinal blood flow when blood pressure drops with sedation because retinal blood flow is associated with the level of intracerebral perfusion. Finally, those eyes with delayed vascular filling times with normal intraocular and systemic blood pressure represent more tenuous circulation and possibly poorer visual prognosis.

Residual vascular abnormalities after membrane delamination of ischemic neovascular retinopathies also represented an important observation. We were able to treat these areas of residual leakage in real time with either laser or diathermy, thereby theoretically reducing the risk of postoperative vitreous hemorrhage. We were also able to preferentially treat regions of ischemic peripheral retina with heavier laser and relatively preserve better perfused areas. The ability to customize the laser pattern in real time intraoperatively will hopefully preserve more peripheral and low light vision compared with indiscriminate panretinal laser placement while reducing the neovascular stimulus by more confluent laser treatment of ischemic retina.

We were also able to better diagnose inflammatory and infectious posterior segment pathologies using IOFA in real time, once the overlying vitreous opacities and inflammation were cleared. We were able to assess the degree of retinal inflammation, cystoid macular edema, and perivascular leakage, which is important when there is a short window of media clarity in eyes that can develop postoperative inflammation.

Intraoperative fluorescein angiography reliably and efficiently reproduced many clinical biomarkers, such as vascular filling times, microvascular abnormalities, and leakage. Taken together with the other advantages of DAVS, IOFA provides access to important fluorescein angiography data to truly enhance surgical visualization during vitrectomies. Moreover, identification of fluorescein angiography biomarkers permitted modification and optimization of many surgical interventions intraoperatively. Thus, IOFA combined with other enhancements to DAVS, such as intraoperative OCT, and the use of artificial intelligence intraoperatively will lead to a significant evolution and refinement to vitreoretinal surgical techniques and outcomes.

## Supplementary Material
